# Association Between Sleep Quality and Academic Performance Among Undergraduate Medical Health Sciences Students: A Cross-Sectional Study

**DOI:** 10.7759/cureus.91548

**Published:** 2025-09-03

**Authors:** Shaimaa Hassan, Najeeb M Alqahtani, Salihah M Alshahrani, Abdulkhalig A Alhefzy, Omar Alharthi, Muhannad Alharbi

**Affiliations:** 1 Histology, General Medicine Practice Program, Batterjee Medical College, Aseer, SAU; 2 General Medicine Practice Program, Batterjee Medical College, Aseer, SAU

**Keywords:** academic performance, medical students, mental health, pittsburgh sleep quality index, saudi arabia, sleep disorders, sleep quality

## Abstract

Background: Adequate sleep is crucial for maintaining memory, emotional regulation, mental sharpness, and general health. Students pursuing medical degrees frequently experience interrupted sleep patterns because of rigorous academic workloads and hospital duties, potentially harming both their wellness and educational outcomes.

Aim: This study aims to examine how sleep quality influences academic performance in medical and health sciences undergraduates at Batterjee Medical College, Saudi Arabia, in the 2024-2025 academic year.

Methods: A cross-sectional study involving 175 medical students was carried out using standardized questionnaires, such as the Epworth Sleepiness Scale and the Pittsburgh Sleep Quality Index (PSQI), to assess sleep quality. The students' academic performance was assessed according to their cumulative grade point average (GPA).

Results: A total of 175 medical students participated in the study, of whom 118 (67.4%) were male. Regarding age distribution, 95 (54.3%) were aged 18-22, and 41 (23.4%) were aged 23-27. The participants came from various specialties, including pharmacy, dentistry, medicine, physical therapy, and nursing, with educational levels ranging from preparatory year to postgraduate. Among the 175 responses received, 93 (53.1%) had an outstanding GPA, 13 (7.4%) had a good GPA, and two (1.1%) had a below-average GPA. The average PSQI score was 6.7 (±3.5). Poor sleep quality was reported by 129 (73.7%) of participants, while 46 (26.3%) reported good sleep quality. Key factors influencing sleep quality included caffeine consumption (157, 89.7%), family history of sleep disorders (38, 21.7%), psychiatric illness (25, 14.3%), and chronic diseases (10, 5.7%).

Conclusion: Poor sleep quality is a common issue among medical students, negatively impacting both their academic success and overall well-being. Implementing effective sleep habits and better time management strategies is crucial for improvement.

## Introduction

Sleep is a fundamental pillar of health, playing a crucial role in mental and physical well-being, cognitive function, and overall quality of life. It is crucial for memory consolidation and learning. However, medical and health science students often experience reduced sleep due to their demanding academic and clinical workloads [1].

Sleep deprivation negatively impacts memory encoding and consolidation, processes vital for medical students, who must absorb a high volume of knowledge throughout their studies [2]. Research indicates that college students who experience insufficient sleep often face challenges in academic achievement and physical health [3]. Additionally, persistent lack of sleep, excessive daytime drowsiness, and regularly sleeping less than seven hours nightly are associated with a higher chance of developing mental health conditions [4].

Studies among Saudi medical students have reported high stress levels and poor sleep quality, contributing to a decline in their overall quality of life. Consequently, medical students are more exposed to impaired physical and emotional well-being [5].

Poor sleep quality or irregular sleep patterns can negatively impact mental health [6]. Research conducted on university students across 16 countries revealed that both insufficient sleep (less than seven hours) and excessive sleep (more than nine hours) were associated with heightened feelings of social isolation. This indicates a U-shaped correlation, where optimal sleep duration is crucial for psychological well-being [7].

Recent studies have extensively examined the connection between perceived sleep quality and academic success. Many have found that students who get adequate, restful sleep tend to achieve higher grades, whereas those with poor sleep quality or sleep deprivation often perform worse academically [8].

Similarly, Esht et al. [9] found that individuals with healthy sleep patterns experience fewer mental health problems and perform better academically compared to those with disrupted sleep. Their research emphasizes that poor sleep quality increases susceptibility to psychological distress and academic difficulties, underscoring the need for interventions to enhance sleep habits.

Multiple factors have been linked to poor sleep quality, such as academic standing, stress levels, physical inactivity, alcohol consumption, drug use, and overuse of smartphones. These elements can help healthcare professionals identify individuals at greater risk of sleep problems. Additional influences, including caffeine consumption, obesity, and obstructive sleep apnea, also play a role in sleep quality [10,11].

Emerging research underscores the strong connection between sleep quality and academic achievement in college students. A study conducted at Menoufia University in Egypt found that an overwhelming majority (96.5%) of medical students had poor sleep quality according to the Pittsburgh Sleep Quality Index (PSQI) assessments. These students slept an average of 6.67 hours per night, and their sleep difficulties were strongly correlated with lower academic scores. Lifestyle habits such as smoking, lack of exercise, and prolonged internet use were also associated with sleep disturbances in this group [12].

A study conducted in Saudi Arabia explored how sleep duration and quality affect academic performance among health sciences students. The results revealed that students who slept six to seven hours nightly achieved higher GPAs than those who slept less or more. Interestingly, no clear link was found between overall sleep quality (measured by the PSQI) and GPA, implying that, while sleep duration may impact academic success, other sleep-related factors could also be influential [1].

Another study from health technology colleges in Akure, Ondo State, Nigeria, investigated the connection between sleep quality and academic achievement. The research showed that fewer than half of the students had good sleep quality, while more than half reported poor sleep. Many students also had lower academic performance, potentially due to habits such as smoking, daytime napping, caffeine intake, and using mobile phones before bed. These behaviors negatively impacted both sleep and grades [13].

These studies emphasize the important role of both sufficient sleep duration and good sleep quality in boosting academic performance among undergraduate students. They also highlight the need for interventions addressing lifestyle factors detrimental to sleep, such as using electronic devices too much before sleep and drinking stimulants such as caffeine. Encouraging students to adopt healthier sleep habits could enhance their academic success and overall well-being. Additionally, these studies underscore the necessity for further research to understand the relationship between sleep quality and academic performance in undergraduate populations.

This cross-sectional study examines the association between sleep quality and academic achievement among medical and health sciences undergraduates at Batterjee Medical College (BMC) in Saudi Arabia. Given the rigorous academic and clinical workload these students encounter, sleep disturbances and insufficient rest are common concerns. Since quality sleep is critical for cognitive performance, memory retention, and general health, poor sleep patterns may negatively impact educational success. Understanding this association can help guide interventions to improve sleep quality, thereby enhancing academic performance and mental health in this vulnerable group.

## Materials and methods

Sampling method

A convenience sampling method was used in this cross-sectional study. Students at BMC who were enrolled in the academic year 2024-2025, across all levels, constituted the study population and were eligible to participate in this study.

Study area

This study was conducted at BMC in Saudi Arabia, focusing on its undergraduate students.

Study subjects

The study subjects were students enrolled at BMC during the academic year 2024-2025 across all levels.

Inclusion and exclusion criteria

The following are the inclusion criteria of the study: (1) undergraduate students who are currently enrolled in any program at BMC for the 2024-2025 academic year; (2) students who are willing to participate and provide informed consent; and (3) students who are able to complete the survey.

The following are the exclusion criteria of the study: (1) students who have been taking medications known to influence sleep patterns, such as alpha-blockers, corticosteroids, glucosamine, chondroitin, statins, or sedative-hypnotics (including barbiturates and benzodiazepines), within the past month prior to data collection; and (2) participants who have severe psychiatric conditions (e.g., active mania or psychosis).

Sample size

The sample size was estimated using the following formula [14]: 

n = \frac{Z^{2} \, P (1 - P)}{d^{2}}

Here, n = sample size, z = confidence level (Z = 1.96 for a 95% confidence level), d = margin of error (precision) = 0.05, and p = prevalence of poor sleep quality among medical students in Saudi Arabia (88%, based on a previous study) [15]. After accounting for a 10% non-response rate, the final adjusted sample size was 178 participants.

Study design

A cross-sectional online questionnaire, self-administered by participants, was used in this study to gather data from students at BMC.

Data collection tool

The questionnaire was divided into four parts. The first section collected sociodemographic data, such as gender, age, education level, body mass index (BMI), any history of chronic or psychiatric conditions, family background of sleep disorders, consumption of coffee or tea, smoking habits, screen time (TV or mobile use), and cumulative GPA, which served as a measure of academic performance.

The second section employed the PSQI, a validated open-access instrument designed to evaluate participants' sleep quality over the past month. According to Buysse et al. [16], the PSQI comprises seven domains: subjective sleep quality, sleep latency, sleep duration, use of sleep medication, sleep efficiency, sleep disturbances, and daytime dysfunction. Each domain is scored from 0 to 3, resulting in a total PSQI score ranging from 0 to 21. A score above 5 indicates poor sleep quality, while a score of 5 or below suggests good sleep quality [16].

The third section utilized the Epworth Sleepiness Scale (ESS), another validated tool used under license, to measure daytime sleepiness levels [17].

Finally, the fourth section incorporated a validated open-access survey assessing sleep disturbances and their impact on academic performance [18].

Data collection process

The study employed an online self-reported questionnaire as the main tool for gathering data. The research team distributed the survey link via popular social networks, such as Twitter and WhatsApp, which were frequented by the target population. Prospective respondents also received a detailed information document explaining the research aims, study goals, and participant requirements before taking part.

Statistical techniques

Statistical analyses were conducted using Statistical Product and Service Solutions (SPSS, version 25.0; IBM SPSS Statistics for Windows, Armonk, NY). Sociodemographic data and questionnaire responses were summarized using descriptive statistics. Continuous variables were presented as mean ± standard deviation, while categorical data were expressed as counts and percentages. The normality of continuous variables was assessed using the Kolmogorov-Smirnov test. Depending on data distribution, either parametric tests (independent samples t-test, ANOVA) or non-parametric alternatives (Mann-Whitney U, Kruskal-Wallis) were used to examine relationships between independent and outcome variables. For categorical outcomes, Pearson’s chi-square test will be applied.

To identify factors influencing poor sleep quality in medical students, multivariate logistic regression was performed. Statistical significance set at p < 0.05. Additionally, academic performance was evaluated by categorizing students into two groups based on GPA (below 2.0 vs. 2.0 or higher) to assess the association with sleep disturbances.

Ethical approval

Written informed consent was obtained from all participants prior to data collection, with full disclosure of the study’s aims, methodology, and potential risks. Ethical approval was secured from the Research Ethics Committee at BMC (reference no. RES-2025-0038), guaranteeing that the study followed rigorous ethical protocols.

## Results

A total of 175 medical students participated in the study. The majority of participants were male (118, 67.4%). Ninety-five participants (54.3%) were between the ages of 18 and 22, while 41 (23.4%) were between 23 and 27. Participants came from diverse specialties, including pharmacy, dentistry, medicine, physical therapy, and nursing, with educational levels ranging from preparatory year to postgraduate.

More than half of the participants (103, 58.9%) had a normal weight, 33 (18.9%) were overweight, and only seven (4%) had class III obesity. Among the 175 responses regarding academic performance, 93 (53.1%) reported an outstanding GPA, 13 (7.4%) had a good GPA, and two (1.1%) had a below-average GPA.

The average PSQI score was 6.7 ± 3.5, with 129 (73.7%) of participants reporting poor sleep quality and 46 (26.3%) reporting good sleep quality. When asked about factors influencing their sleep quality, participants cited the following: caffeine (157, 89.7%), family history of sleep disorders (38, 21.7%), psychiatric illness (25, 14.3%), and chronic diseases (10, 5.7%) (Table 1).

**Table 1:** Demographic and sleep characteristics of the participants (N=175) BMI: body mass index; PSQI: Pittsburgh Sleep Quality Index

Characteristics	Categories	N	%
Age in years	18-22	95	54.3
	23-27	41	23.4
	28-30	13	7.4
	31-35	14	8
	36-40	12	6.9
Gender	Male	118	67.4
	Female	57	32.6
Disciplines	Preparatory year	36	20.6
	Medicine	70	40
	Health care administration	26	14.9
	Respiratory therapy	3	1.7
	Nursing	9	5.1
	Dentist	25	14.3
	Pharmacy	4	2.3
	Physical therapy	1	0.6
	Nutrition	1	0.6
Current year of study	Preparatory year	12	6.9
	1st year	61	34.9
	2nd year	16	9.1
	3rd year	31	17.7
	4th year	19	10.9
	5th year	19	10.9
	Intern	13	7.4
	Postgraduate	4	2.3
Current GPA	Below average	2	1.1
	Good	13	7.4
	Very good	30	17.1
	Excellent	37	21.1
	Outstanding	93	53.1
Factors that affect sleep quality	Chronic diseases	10	5.7
	Psychiatric illness	25	14.3
	Family history of sleep disorders (e.g., insomnia, sleep apnea)	38	21.7
	Caffeine	157	89.7
	Smoker	30	17.1
BMI categories in kg/m^2^	Underweight	19	10.9
	Normal weight	103	58.9
	Overweight	33	18.9
	Obesity class1	7	4
	Obesity class 2	6	3.4
	Obesity class3	7	4
PSQI total score	(Mean ± SD)	6.7 (3.5)	
During the past month, hours of actual sleep	(Mean ± SD)	6.2 (2.1)	
PSQI categories	Poor sleep quality	129	73.7
	Poor sleep quality	46	26.3
Sleep latency	More than or equal to 30 min	70	40
	Less than 30 min	105	60

Among the 175 participants, 76 (43.4%) reported having fairly good sleep quality, while 29 (16.6%) reported fairly bad sleep quality when rating their sleep over the past month. Regarding factors causing sleep disturbances three or more times per week in the past month, participants reported the following: having to get up to use the bathroom (18, 10.3%), being unable to breathe comfortably (12, 6.9%), coughing or snoring loudly (7, 4%), feeling too cold (13, 7.4), feeling too hot (8, 4.6), having bad dreams (8, 4.6%), and experiencing pain (12, 6.9%) (Tables 2-5).

**Table 2:** A - Factors influencing sleep patterns in the month prior to responding to the study (N=175)

5. During the past month, how often have you had trouble sleeping because you…	Not during the past month, N (%)	Less than once a week, N (%)	Once or twice a week, N (%)	Three or more times a week, N (%)
a. Cannot get to sleep within 30 min	66 (37.7)	44 (25.1)	32 (18.3)	33 (18.9)
b. Wake up in the middle of the night or early morning	70 (40)	51 (29.1)	24 (13.7)	30 (17.1)
c. Have to get up to use the bathroom	78 (44.6)	56 (32)	23 (13.1)	18 (10.3)
d. Cannot breathe comfortably	105 (60)	42 (24)	16 (9.1)	12 (6.9)
e. Cough or snore loudly	123 (70.3)	28 (16)	17 (9.7)	7 (4)
f. Feel too cold	92 (52.6)	47 (26.9)	32 (13.1)	13 (7.4)
g. Feel too hot	93 (53.1)	48 (27.4)	26 (14.9)	8 (4.6)
h. Have bad dreams	88 (50.3)	61 (34.9)	18 (10.3)	8 (4.6)
i. Have pain	110 (62.9)	43 (24.6)	10 (5.7)	12 (6.9)
6. During the past month, how often have you taken medicine to help you sleep (pre­scribed or “Over the counter”)?	119 (68)	38 (21.7)	12 (6.9)	6 (3.4)
7. During the past month, how often have you had trouble staying awake while driving, eating meals, or engaging in social activity?	92 (52.6)	57 (32.6)	20 (11.4)	6 (3.4)

**Table 3:** B - Factors influencing sleep patterns in the month prior to responding to the study (N=175)

8. During the past month, how much of a problem has it been for you to keep up enough enthusiasm to get things done?	No problem at all	Only a very slight problem	Somewhat of a problem		A very big problem
	55 (31.4)	69 (39.4)		34 (19.4)	17 (9.7)

**Table 4:** C - Factors influencing sleep patterns in the month prior to responding to the study (N=175)

9. During the past month, how would you rate your sleep quality overall?	Very good	Fairly good	Fairly bad		Very bad
	58 (33.1)	76 (43.4)		29 (16.6)	12 (6.9)

**Table 5:** D - Factors influencing sleep patterns in the month prior to responding to the study (N=175)

10. Do you have a bed partner or room Mate?	No bed partner or room mate	Partner/roommate in other room	Partner in same room but not same bed	Partner in same bed
	80 (45.7)	29 (16.6)	43 (24.6)	23 (13.1)

**Figure 1:**
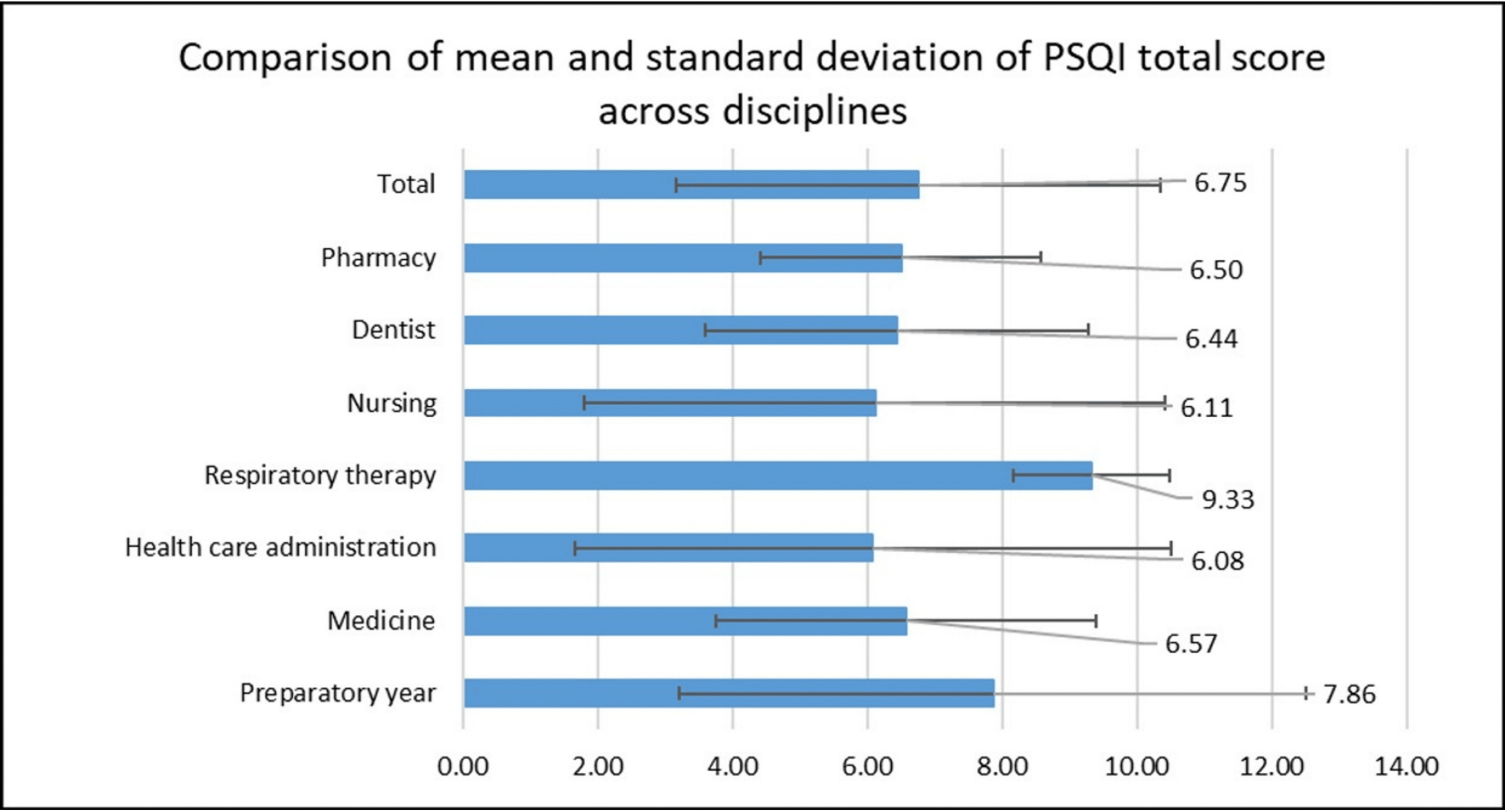
Comparison of mean and standard deviation of the PSQI total score across disciplines PSQI: Pittsburgh Sleep Quality Index

Figure 1 displays the average scores for each PSQI component and the total sleep quality scores across various disciplines. The total sleep quality scores are similar among disciplines, indicating that overall sleep quality is relatively consistent across student groups. Minor variations exist, with respiratory therapy students showing the highest mean score (9.3) and healthcare administration students the lowest (6.08). However, these differences are minimal, suggesting little significant variation between disciplines.

**Figure 2:**
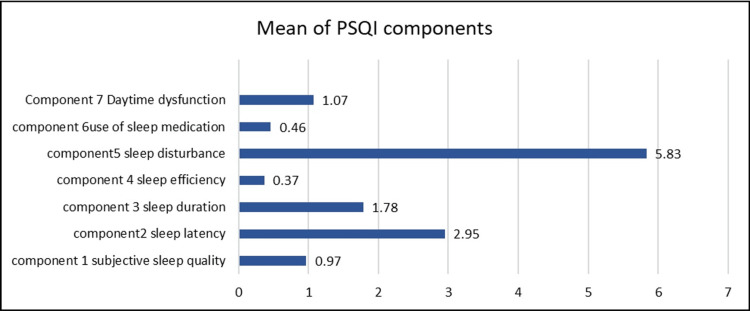
Mean of PSQI components PSQI: Pittsburgh Sleep Quality Index

Figure 2 presents the mean scores of individual PSQI components, showing that sleep disturbance (Component 5) had the highest average (5.83), while sleep efficiency (Component 4) had the lowest (0.37).

As presented in Table 6, the frequency of reported sleep disturbances varies among individuals sharing a bed or room. The most frequently reported issue was disorientation or confusion during sleep, experienced by eight participants. This was followed by leg twitching or jerking during sleep, reported by six individuals. Five participants noted other forms of restless sleep, while only one individual reported long pauses in breathing during sleep, making it the least common disturbance.

**Table 6:** The prevalence of sleep disturbances among individuals sharing a bedroom or bed (N=175)

Sleep Disturbances	Not during the past month, N (%)	Less than once a week, N (%)	Once or twice a week, N (%)	Three or more times a week, N (%)
Loud snoring	124 (70.9)	36 (20.6)	11 (6.3)	4 (2.3)
Long pauses between breaths while asleep	131 (74.9)	32 (18.3)	11 (6.3)	1 (0.6)
Legs twitching or jerking while you sleep	120 (68.6)	34 (19.4)	15 (8.6)	6 (3.4)
Episodes of disorientation or confusion during sleep	111 (63.4)	42 (24)	14 (8)	8 (4.6)
Other restlessness while you sleep, please describe	120 (68.6)	35 (20)	15 (8.6)	5 (2.9)

As illustrated in Table 7, 46 (80.7%) of female participants reported poor sleep quality. Additionally, over 90% of individuals aged 28-35 experienced sleep disturbances. Sleep quality varied significantly based on living arrangements: those sharing a room with a partner but sleeping separately (26, 89.7%) had worse sleep compared to those without a roommate (49, 61.3%) (*p* = 0.003). A notable difference was also observed between students sharing a room (but not a bed) with a partner and those living alone.

**Table 7:** Predictors of sleep quality among study participants • Fisher's exact test

Factors	Poor sleep quality (N=129)	Good sleep quality (N=46)	Bivariate		Multivariate regression	
	N (%)	N (%)	X^2^	P value	OR (CI)	P value
Gender Male	83 (70.3)	35 (29.7)	2.130	0.144	Reference	
Female	46 (80.7)	11 (19.3)			0.43 (0.1-1.0)	0.073
Age 18-22	68 (71.6)	27 (28.4)	6.06•	0.190	2.7 (0.3-25)	0.36
23-27	28 (68.3)	13 (31.7)			1.9 (0.2-14)	0.49
28-30	12 (92.3)	1 (7.7)			9.2 (0.5-14)	0.11
31-35	13 (92.3)	1 (7.1)			16.7 (1.1-23)	0.03
36-40	8 (66.7)	4 (33.3)			Reference	-
Academic year Preparatory year	10 (83.3)	2 (16.7)	7.541^•^	0.359	Reference	-
2nd year	11 (68.8)	5 (31.3)			0.4 (0.08-2)	0.28
3rd year	26 (83.9)	5 (16.1)			0.2 (0.03-1.6)	0.14
4th year	11 (57.9)	8 (42.1)			0.8 (0.1-5)	0.85
5th year	16 (84.2)	3 (15.8)			0.2 (0.03-1.4)	0.11
Intern	9 (69.2)	4 (30.8)			0.9 (0.1-6.7)	0.95
Postgraduate	4 (100)	0			0.3 (0.05-2.7)	0.34
GPA in last exam Below average	2 (100)	0	2.127^•^	0.708	-	-
Good	11 (84.6)	2 (15.4)			2.2 (0.4-10)	0.31
Very good	24 (80)	6 (20)			1.6 (0.6-4.4)	0.33
Excellent	26 (70.3)	11 (29.7)			0.9 (0.4-2.2)	0.93
Outstanding	66 (71)	27 (29)			Reference	-
Bed partner No bed partner or room mate	49 (61.3)	31 (38.8)	13.593	0.003	Reference	-
Partner/roommate in other room	26 (89.7)	3 (10.3)			2.9 (0.5-15)	0.19
Partner in same room but not same bed	37 (86)	6 (14)			3.8 (1.3-11)	0.01
Partner in same bed	17 (73.9)	6 (26.1)			1.5 (0.3-6)	0.53

Academic performance also influenced sleep quality, with all participants who scored below average on their last exam (100%) reporting disrupted sleep, followed by 11 (84.6%) of those who achieved good grades. Multivariate logistic regression revealed significantly higher odds of poor sleep among medical students aged 31-35 (OR = 16.7; 95% CI, 1.1-23; *p* = 0.03). However, gender, academic year, and GPA did not show a statistically significant association with insufficient sleep in the regression analysis.

## Discussion

The results of this study reveal a significant and pervasive issue affecting medical and health sciences students at BMC: poor sleep quality. With 73.7% of participants reporting poor sleep, it is evident that this is not a minor or isolated concern but rather a systemic challenge embedded within the educational environment of future healthcare professionals. Although the multivariate analysis did not show statistically significant correlations between sleep quality and GPA, the directional trends, combined with supporting evidence from other studies, suggest that poor sleep likely undermines academic performance, cognitive function, and overall well-being.

Sleep is fundamentally tied to learning, memory consolidation, emotional regulation, and attention, factors crucial to student success, particularly in demanding academic settings such as medical colleges [19]. The average PSQI score of 6.7 among participants surpasses the diagnostic threshold for poor sleep (PSQI > 5), indicating chronic sleep issues that warrant immediate attention. Notably, all students with "below average" GPAs (1.1% of respondents) reported poor sleep, while a majority of those with "outstanding" GPAs still faced sleep problems. This suggests a spectrum of impact, with poor sleep being prevalent across performance levels but most detrimental to those already academically vulnerable.

Our findings align with a growing body of research across diverse countries and academic contexts. For example, Shehata et al. [12] conducted a cross-sectional study on Egyptian medical students at Menoufia University and found that 96.5% had poor sleep quality, which was directly associated with lower academic performance, excessive daytime sleepiness, and reduced concentration. Notably, students who slept fewer than six hours per night or experienced frequent sleep disturbances performed worse academically than their well-rested peers. This mirrors the pattern observed in our study, where even high-achieving students showed signs of sleep disruption, but the most adverse effects were concentrated among lower achievers.

Similarly, Alotaibi et al. [20] found that Jordanian medical students with poor sleep quality were twice as likely to fail or underperform in their academic evaluations. Their study emphasized the role of environmental factors - such as excessive smartphone use, late-night study habits, and lack of physical activity, variables that were not directly measured in our study but likely contribute to the high prevalence of poor sleep we observed. The consistency of these findings across different regional contexts highlights a universal pattern among medical students globally, pointing to shared stressors and lifestyle habits characteristic of medical education [20].

An important dimension revealed in our study is the role of lifestyle and environmental factors. A significant proportion of participants reported caffeine use (89.7%), psychiatric illness (14.3%), and a family history of sleep disorders (21.7%) as factors influencing their sleep. These findings align with existing literature. For instance, Maheshwari et al. [21] found that stimulant consumption (particularly caffeine and energy drinks), as well as pre-bedtime smartphone use, was directly associated with delayed sleep onset, fragmented sleep, and daytime fatigue among medical students in South Asia. Ironically, the use of stimulants to combat academic fatigue appears to fuel a vicious cycle, delaying sleep and further exacerbating the exhaustion that initially prompted stimulant use [21].

Additionally, our findings highlight social and demographic influences on sleep quality. Students who shared a room with a partner (but not a bed) were significantly more likely to report poor sleep quality. This supports the view proposed by Hirshkowitz et al. [22], who argued that shared living environments, particularly those with inconsistent schedules and noise exposure, disrupt circadian rhythms and reduce perceived sleep satisfaction. Moreover, although not statistically significant, female students reported higher rates of poor sleep (80.7%) than their male counterparts (70.3%). Gender differences in sleep patterns and susceptibility to insomnia are well-documented, often attributed to hormonal fluctuations, greater stress sensitivity, and the higher prevalence of anxiety and depression among female students [23].

One particularly interesting comparison comes from Fonseca et al. [24], who suggest that academic performance is linked not only to how much students sleep but also to how well and when they sleep. Their model, the “3Q model” (quality, quantity, and timing), posits that students who go to bed late, even if they sleep for seven to eight hours, may still experience impaired cognitive function due to disrupted circadian alignment. Although our study did not measure bedtime or chronotype, the high prevalence of sleep disturbances (e.g., frequent awakenings, difficulty falling asleep, and use of sleep medications) suggests that timing and quality are significant issues warranting further research [24].

Although our multivariate logistic regression analysis did not identify GPA as a significant independent predictor of sleep quality, this does not negate its potential impact. The complex interplay between academic stress, sleep disruption, and mental health may mediate this relationship. Students with high GPAs might still sacrifice sleep to meet performance expectations, whereas those lacking coping mechanisms or strong academic foundations may be more vulnerable to the cumulative effects of sleep deprivation. Future longitudinal studies should explore the bidirectional nature of this relationship, where poor sleep impairs academic performance and academic stress further disrupts sleep [25].

Importantly, our findings underscore the need for targeted institutional interventions. Universities and medical colleges should recognize sleep not merely as a personal health issue but as a critical determinant of academic success and mental resilience. Initiatives such as sleep education workshops, mindfulness programs, reduced nighttime academic workloads, and improved access to counseling services could help break the cycle of poor sleep and suboptimal performance. Additionally, flexible scheduling, particularly during high-stress periods such as exam weeks, could help students align their natural sleep patterns with academic demands.

Finally, the findings raise concerns about the long-term health consequences of chronic poor sleep among future healthcare professionals. Medical students are tomorrow’s physicians, and if they graduate with health compromised by years of sleep deprivation, the repercussions extend beyond their personal well-being to the quality of care they provide. As Genzel et al. [19] aptly noted, “We must first care for the caregivers, beginning in medical school.”

Limitations

This study employed a cross-sectional approach, making it difficult to determine cause-and-effect relationships between sleep quality and academic achievement. Because the data were gathered during one specific period, they might not fully represent lasting patterns or demonstrate direct causal connections. Data on sleep quality and lifestyle factors were obtained through self-administered questionnaires, such as the PSQI and ESS. This method introduces potential biases, including recall bias, social desirability bias, and misreporting.

Additionally, the sample was limited to undergraduate students from a single institution (BMC, Saudi Arabia), which may restrict the generalizability of the findings to other student populations or universities with different academic pressures or cultural contexts. Although demographic and lifestyle factors were assessed, residual confounders, such as undiagnosed sleep disorders, mental health conditions, and socioeconomic status, may not have been fully accounted for in the analysis, potentially influencing the observed associations.

Furthermore, excluding students who used sleep-affecting medications or had psychiatric conditions may have improved internal validity. However, this exclusion could reduce external validity by omitting a subgroup of the population that often experiences poor sleep quality.

## Conclusions

The present study confirmed that poor sleep quality is a widespread and significant issue among medical students at BMC, affecting students across different GPA levels, though the multivariate analysis did not reveal statistically significant correlations between sleep quality and GPA. Based on these findings, several institution-level recommendations are proposed to help mitigate the negative effects of sleep disturbances, such as incorporating sleep health and time management training into medical curricula and reforming academic schedules to align with students’ natural circadian rhythms, particularly during high-stress periods. A more detailed investigation is necessary to identify the factors influencing sleep quality and how they impact academic performance. Additionally, increasing awareness about healthy sleep practices and highlighting the positive effects of quality sleep on both physical and mental well-being is essential.
